# Editorial: Congenital craniofacial deformities: genetic and clinical aspects

**DOI:** 10.3389/froh.2023.1298447

**Published:** 2023-10-05

**Authors:** Hanyao Huang, Juan Du

**Affiliations:** ^1^ State Key Laboratory of Oral Diseases and National Clinical Research Center for Oral Diseases and Department of Oral Maxillofacial Surgery, West China Hospital of Stomatology, Sichuan University, Chengdu, China; ^2^ Laboratory of Orofacial Development, Laboratory of Molecular Signaling and Stem Cells Therapy, Molecular Laboratory for Gene Therapy and Tooth Regeneration, Beijing Key Laboratory of Tooth Regeneration and Function Reconstruction, Capital Medical University School of Stomatology, Beijing, China

**Keywords:** congenital craniofacial deformities, cleft lip and palate (CLP), craniofacial abnormalities, oral health, velopharyngeal (VP) insufficiency

**Editorial on the Research Topic** Congenital craniofacial deformities: genetic and clinical aspects

The Research Topic “Congenital Craniofacial Deformities: Genetic and Clinical Aspects” is a Frontiers Research Topic aimed to provide an opportunity for researchers and clinicians from different perspectives and areas to publish recent advances in investigating the genetic etiology and the treatment options for different congenital craniofacial deformities.

Caused by genetic diseases, chromosomal mutations, or abnormal embryonic development, congenital craniofacial deformities refer to the inborn anomalies of the skull, orbits, zygomatic bones, maxilla, and mandible. Facial soft tissue defects, including craniosynostosis, craniofacial fissure, dilated orbital distance, and craniofacial microscopic anomalies are also considered craniofacial deformities. In severe cases, they can even lead to intellectual disability and visual impairment. Craniofacial deformities are often accompanied by anomalies of the spine, trunk, limbs, and visceral transposition. The treatment of congenital craniofacial deformities is complex, and satisfying outcomes are still missing. Furthermore, the patient's psychological state and related social behavior also need more attention. Even though a lot of research has been carried out in the field of craniofacial development, the genetic etiology of congenital craniofacial deformities needs more investigation to reveal the role of specific genes and their synergistic effect during the development of the craniofacial skeletal system.

Cleft lip and palate is one of the most common congenital craniofacial deformities. For repairing cleft lip and palate, team approach should be carried out. Restorations of primary deformities and secondary dysfunctions are the main purposes in the treatment of cleft lip and palate.

In this Research Topic, presurgical intervention of primary cleft lip repair is focused by Yin et al. Outcomes by three dimensional evaluation of presurgical nasoalveolar molding (PNAM) therapy in patients with non-syndromic complete unilateral cleft lip and palate (UCLP) were presented. Their results demonstrated that PNAM therapy could help decrease the difficulties of cleft lip repair.

Rhinoplasty for patients with cleft lip is always a challenge to the surgeons, and narrow nostril deformities are the most troublesome. The study by Wei et al. developed an algorithm for surgical method selection for repairing narrow nostrils in patient with secondary cleft lip nasal deformities. It demonstrated that the width of the nasal floor and the length of the alar rim are critical elements for selecting the appropriate surgical method. Meanwhile, a review by Xu et al. focusing on the growth patterns of the nasolabial region following unilateral cleft lip primary repair was also included in this Research Topic.

The purpose of cleft palate repair is to reconstructing a normal velopharyngeal function of the patient. In this Research Topic, the study by Fan et al. compared the velopharyngeal morphology of patients with hard and soft cleft palate after Furlow and Sommerlad palatoplasty, and Mao et al. presented a review to introduce specific problems that are currently under-recognized in the diagnosis and treatment of marginal velopharyngeal inadequacy and provides guidelines for further exploration of standardized and reasonable intervention protocols for marginal velopharyngeal inadequacy.

A review about poor oral hygiene and high susceptibility to dental caries and periodontitis in patients with cleft lip and palate was presented by Wu et al. which concluded and updated probable causes underlying the association between cleft lip and palate and poor oral health.

Meanwhile, application of vertical transposition flap in closure for large facial soft tissue defects in children was reported by Feng et al. A case with hereditary sensory autonomic neuropathy was also reported by O’hagan-Wong et al. which demonstrated extensive oral trauma as one of the early signs of hereditary sensory autonomic neuropathy that should provoke a timely referral for neurological assessment.

This Research Topic targets the acquisition and dissemination of knowledge regarding to congenital craniofacial deformities to share knowledge from both the basic and clinical sciences. Briefly, this Research Topic provided researchers and clinicians from different perspectives and areas with a platform to discuss recent advances in the understanding of congenital craniofacial deformities. It also will provide readers with new insights and different viewpoints to stimulate further investigations in this broad research field.

